# HIV Infection Is Not Associated with Carotid Intima-Media Thickness in Brazil: A Cross-Sectional Analysis from the INI/ELSA-Brasil Study

**DOI:** 10.1371/journal.pone.0158999

**Published:** 2016-07-08

**Authors:** Antonio G. Pacheco, Beatriz Grinsztejn, Maria de Jesus M. da Fonseca, Rosane Härter Griep, Paulo Lotufo, Isabela Bensenor, José G. Mill, Rodrigo de C. Moreira, Ronaldo I. Moreira, Ruth K. Friedman, Marilia Santini-Oliveira, Sandra W. Cardoso, Valdiléa G. Veloso, Dóra Chor

**Affiliations:** 1 FIOCRUZ, Programa de Computação Científica, Rio de Janeiro, Brazil; 2 FIOCRUZ, Instituto Nacional de Infectologia Evandro Chagas, Rio de Janeiro, Brazil; 3 FIOCRUZ, Departamento de Epidemiologia e Métodos Quantitativos em Saúde, Rio de Janeiro, Brazil; 4 FIOCRUZ, Laboratory of Health and Environment Education, Rio de Janeiro, Brazil; 5 University of São Paulo, School of Medicine, São Paulo, Brazil; 6 Federal University of Espírito Santo, Departamento de Ciências Fisiológicas, Vitória, Brazil; Azienda ospedaliero-universitaria di Perugia, ITALY

## Abstract

**Background:**

Carotid intima-media thickness (cIMT) has been used as an early marker of atherosclerotic disease in the general population. Recently its role among HIV-infected patients has been questioned. To date, no Brazilian study has compared cIMT in respect to HIV status.

**Methods:**

We compared data from 535 patients actively followed in a prospective cohort in Rio de Janeiro (HIV group); 88 HIV-negative individuals who were nominated by patients (friend controls–FCs); and 10,943 participants of the ELSA-Brasil study. Linear regression models were used to study associations of the 3 groups and several covariables with cIMT. Propensity scores weighting (PSW) were also employed to balance data.

**Results:**

Median thickness in mm (IQR) were 0.54 (0.49,0.62); 0.58 (0.52,0.68); and 0.57 (0.49,0.70), HIV, FCs and ELSA-Brasil groups, respectively (p-value<0.001). The best linear model chosen did not include the group variables, after adjusting for all the variables chosen, showing no difference of cIMT across groups. Similar results were obtained with PSW. Several traditional CVD risk factors were also significantly associated with cIMT: female gender, higher education and higher HDL were negatively associated while risk factors were older age, current/former smoker, AMI/stroke family history, CVD history, hypertension, DM, higher BMI and total cholesterol.

**Conclusions:**

We show for the first time in a middle-income setting that cIMT, is not different in HIV-infected patients in Rio de Janeiro compared with 2 different groups of non-HIV-infected individuals. Traditional CVD risk factors are associated with this outcome. Our results point out that high standards of care and prevention for CVD risk factors should always be sought both in the HIV-infected and non-infected populations to prevent CVD-related events.

## Introduction

After the widespread use of combination antiretroviral therapy (cART) there was a marked shift on the human immunodeficiency virus (HIV)/acquired immunodeficiency syndrome (AIDS) disease profile in terms of morbidity and mortality, including a dramatic increase in patients’ life-expectancy. While the pre-cART era was marked by conditions closely related to several degrees of immunodeficiency as leading causes of death, the current situation shows a more diverse mortality profile [1, 2]. Even though immunodeficiency-related diseases are still the major causes of death [3], there was an important rise in non-AIDS-related conditions, such as malignancies and end-stage liver, renal and cardiovascular diseases [1, 4, 5], which has also been observed in Brazil [2, 3, 6].

As the disease approaches the status of a manageable chronic condition, other risk factors, such as eating habits physical activity, alcohol use, treatment of hypertension, diabetes, socio-demographic characteristics and the effects of aging start to play a more important role for the health status in this population. In addition, chronic exposure to medication [7–11], higher prevalence of smoking [12], metabolic syndrome [13] and HCV-coinfection [14] are other important factors that could be linked to increased risk of CVD in this population.

Carotid intima-media thickness (cIMT) has been used as an early marker of atherosclerotic disease in the general population [15] and in HIV-infected patients [11, 16, 17]. Even though early studies pointed to an association between HIV infection and cIMT thickness [16], recent studies have been showing that this association may not be true, as discussed in a recent review [18]. Very few data have been published to date in low and middle income countries showing the association between HIV infection and cIMT. Studies in Brazil have shown the association between some risk factors and cIMT [19, 20]. We have recently shown that traditional risk factor are more relevant than HIV-specific ones for increased cIMT in a cohort of HIV-infected patients in Rio de Janeiro [21]. Nevertheless, to our knowledge, no Brazilian study has compared cIMT in respect to HIV status.

In this study we compare cIMT in a cohort of HIV-infected patients in Rio de Janeiro, and two other samples: an HIV-negative group and the ELSA-Brasil study.

## Methods

We compared data from three different groups of individuals in a cross-sectional design. The first group was a sample of 649 patients actively followed in the Instituto Nacional de Infectologia Evandro Chagas (INI) prospective cohort in Rio de Janeiro, now referred to as the HIV group [21]. The second group, referred as friend controls–FCs, consisted of 106 individuals who were HIV-negative and were nominated by some of the patients. Friends were nominated by the patient to the study assistant at the day the patient underwent the main study procedures. The third group was the 15,105 participants of the Brazilian Longitudinal Study of Adult Health (ELSA-Brasil study). Data for the ELSA-Brasil group was collected from August 2008 through December 2010, whereas for the other two groups between January 2011 and January 2012. HIV status was not known for the ELSA-Brasil group, but all friend controls were confirmed HIV-negative at the time of data collection.

The study was approved by the Comitê de Ética em Pesquisa from INI, the local institutional review board.

Participants of the 3 groups underwent the ELSA-Brasil study procedures; Elsa-Brasil is a large prospective multicenter cohort of Brazilian civil servants whose main objectives are to study diabetes and cardiovascular disease outcomes and their risk factors in adults; full description of the study procedures can be found elsewhere [22]. The specific study procedures were described in detail in a previous paper [21], and are briefly described as follows: after signing an informed consent form, a questionnaire was applied during an interview at the Investigation Center. A fasting venous blood sample was collected and sent to a central laboratory where all blood tests were performed. A thorough physical examination was performed, comprising anthropometry, resting blood pressure and imaging tests including cIMT measurements [23, 24].

Several variables were also defined or calculated. Race was dichotomized into blacks (including those self-defined as black or mixed black/white race) and non-blacks. Diabetes mellitus (DM) was defined as having glycated hemoglobin >6.5%, fasting glucose > 126 mg/dL, oral glucose tolerance test levels > 200 mg/dL or current use of hypoglycemic drug. Resting blood pressure was measured three times in the non-dominant arm in the sitting position and hypertension was defined if the mean of the last 2 measurements showed systolic blood pressure ≥ 140 mmHg or diastolic blood pressure ≥ 90 mmHg or the use of anti-hypertensive drugs. Dyslipidemia was defined as low density lipoprotein (LDL) levels ≥ 130 mg/dL or use of lipid-lowering drugs. Cumulative smoking was calculated as pack-years, by multiplying the number of packs smoked per day by the number of years of smoking. Family history of sudden death and family history of acute myocardial infarction (AMI) or stroke were defined as the informed occurrence of any episode of those conditions among parents or brothers younger than 65 years of age. History of CVD was defined as informed past occurrence of angina, AMI or stroke. Body mass index (BMI) was calculated as the ratio of measured weight in kilograms and squared height in meters. Cumulative viral load (VL; viremia copy-years) was defined as the area under the curve for two consecutive viral load measurements over time, in years, as described elsewhere [25]. Estimated Glomerular filtration rate (eGFR) was calculated with the CKD-Epi formula [26] taking into account serum creatinine, age, sex and race. A variable to capture poor clinical management was defined as follows: if a participant or patient reported no history of and was taking no medication for hypertension, DM or dyslipidemia and was diagnosed with any of those conditions at the time of entry in the study, we considered that he/she had poor management of those conditions, denoting lack of access to health care.

The main outcome, cIMT, was measured by carotid ultrasonography of 1-cm portions of the distal left and right common carotid artery far walls with a linear transducer (nominal center transducer frequency of 7.5 MHz) (Aplio XG) with axial resolution of approximately 0.10 mm, and calculated automatically by Medical Imaging Applications software (MIA, Coralville, Iowa) over 3 consecutive cardiac cycles [23, 24]. These procedures followed the Mannheim consensus [27]. The average of measurements were used for each artery side and then the average for the left and right arteries was used as the outcome for those participants who had a good quality result for both carotid sides.

General descriptions of variables were performed in respect to the 3 groups and compared with Kruskal-Wallis tests for continuous and chi-squared or Fisher exact tests for categorical variables.

Linear regression models were used to study the association of cIMT with several covariables in the 3 groups. In order to choose the best model explaining cIMT values we used an exhaustive procedure implemented with a genetic algorithm [28], including first-order interactions. In order to avoid possible local best models, which would preclude the selection of the overall best model, 20 replicates were run and the consensus best model was chosen. Model fitness was inspected through residual analysis, which showed that all the models used were adequate.

Model adjustment was also made through a propensity score strategy. Among the several approaches to control for imbalances in group comparisons, we opted to use the non-parametric generalized boosted regression modeling (GBM) [29], which has been described as a very efficient method and less prone to model misspecification [30].

Average treatment effect (ATE) was assumed, in the sense that HIV “exposure” would be the treatment group and the other two groups were considered control groups. The goal was to compare the difference of effect, as measured by the cIMT between those groups. To conform to that jargon, we will refer to the HIV-infected group as “treatment” and non-HIV-infected groups as “control”. A crucial step for this approach is to adequately choose the variables that will be used to calculate the propensity scores [30]. In order to achieve this selection, we used the same variables that were selected by the genetic algorithm to fit the best linear model as described above. Those variables were then used to calculate the propensity scores and weights with GBM.

Balance was assured through usual graphical methods and balance tables to determine if weighted characteristics actually balanced the data between controls and treatment groups. A Kolmogorov-Smirnoff test was employed to assure that weighted characteristics did not statistically differ between treatment and control groups [31]. All variables used reached balance, i.e. all had p-value>0.05.

To establish the association between HIV infection and cIMT, or its lack thereof, we employed several strategies, as described below. First, we conducted two separate analyses, one comparing the HIV group with the friend controls and another comparing it with the ELSA-Brasil group. For each analysis we present 4 models for comparison: 1. a naïve univariable model, for reference; 2. a full model with all the covariables chosen by the genetic algorithm, described above, which yielded different sets of variables for each comparison; 3. a univariable propensity-scores weighted model; 4. a full model with the same variables used to calculate the propensity scores, in order to control for residual confounding.

All analyses were performed with the R environment version 3.2.4 [32].

## Results

Data was available for 649, 106 and 15,105 from the HIV, FCs and ELSA-Brasil groups, respectively. Of those, 535 (82%); 88 (83%) and 10,943 (72%) underwent cIMT measurements and had adequate image quality for analysis; median thickness in mm (IQR) were 0.54 (0.49,0.62); 0.58 (0.52,0.68); and 0.57 (0.49,0.70), respectively (p-value <0.001). Table 1 shows overall comparisons between the 3 groups. Several other variables were significantly different, including gender, age, income, education, and other CVD risk factors. It is of note that the HIV group presented a smaller median cIMT compared to the other two groups and that in general the 3 groups were very different concerning the variables studied. In terms of variables known to be associated with increased risk of CVD and cIMT, the HIV group had more males and blacks, more current smokers and higher amount of cumulative pack-years, more family history of sudden death and history of CVD, lower HDL, higher triglycerides, and higher eGFR, compared to the other two groups. On the other hand, the HIV group was younger, had lower LDL, waist circumference, BMI and C-reactive protein than the other two groups. Table 2 depicts the main characteristics of the HIV group by cART use. Of the 535 patients with cIMT results, 476 (89%) were on cART, with a median use of 4.64 years. CD4 at the time of study entry was similar in both cART users and cART naïve patients, but the latter had significant higher nadir values.

**Table 1:** Characteristics of participants stratified by groups^a^.

		*Groups*			
	ELSA	Friend Controls	HIV	Total	P value
**Total**	10943	88	535	11566	
**Sex: Male**	4880 (44.59)	31 (35.23)	306 (57.2)	5217 (45.11)	< 0.001
**Age in years—median(IQR)**	51 (45,58)	44.48 (36.62,54.61)	43.59 (36.34,51.01)	51 (45,58)	< 0.001
**Schooling: <9 years**	1409 (12.88)	51 (57.95)	259 (48.41)	1719 (14.86)	< 0.001
**Black Race: Yes**	4349 (40.19)	55 (62.5)	341 (63.74)	4745 (41.47)	< 0.001
**Smoking**					< 0.001
**Never**	6214 (56.79)	51 (57.95)	271 (50.65)	6536 (56.52)	
**Former**	3271 (29.89)	20 (22.73)	142 (26.54)	3433 (29.68)	
**Current**	1457 (13.32)	17 (19.32)	122 (22.8)	1596 (13.8)	
**Packs.year (10+)**	2577 (23.66)	20 (22.73)	143 (26.78)	2740 (23.8)	0.2481
**Income (R$)—median(IQR)**	1347.12 (725.38,2279.75)	414.5 (207.25,656.29)	414.5 (207.25,1036.25)	1312.58 (690.83,2279.75)	< 0.001
**Family History of Sudden Death**	1652 (15.1)	11 (12.5)	105 (19.63)	1768 (15.29)	0.0135
**Family History of AMI or Stroke**	2939 (26.86)	31 (35.23)	148 (27.66)	3118 (26.96)	0.1972
**History of CVD**	575 (5.25)	7 (7.95)	38 (7.1)	620 (5.36)	0.0997
**Total cholesterol (mg/dL)—median(IQR)**	211 (186,239)	186.5 (162,214.25)	182 (156,210)	210 (184,238)	< 0.001
**HDL (mg/dL)—median(IQR)**	54 (46,65)	44.5 (39,53)	42 (35,52)	54 (46,65)	< 0.001
**LDL (mg/dL)—median(IQR)**	128 (107,152)	115 (96.75,139)	106 (87,133)	128 (106,151)	< 0.001
**Triglycerides (mg/dL)—median(IQR)**	113 (80,162)	98 (68.5,135)	120 (85,185)	113 (81,163)	< 0.001
**Dyslipidemia**	6315 (57.73)	35 (39.77)	199 (38.05)	6549 (56.7)	< 0.001
**Use of lipid-lowering drugs**	1417 (12.95)	6 (6.82)	74 (13.83)	1497 (12.94)	0.1916
**Hypertension**	3936 (35.97)	37 (42.05)	168 (31.4)	4141 (35.8)	0.0466
**DM**	1905 (17.41)	30 (34.09)	131 (24.49)	2066 (17.86)	< 0.001
**Waist circumference (cm)- median(IQR)**	89.9 (81.5,98.5)	90 (84,98.53)	85.7 (78,94.05)	89.7 (81.4,98.3)	< 0.001
**BMI—median(IQR)**	26.43 (23.81,29.55)	27.51 (24.13,30.1)	24.35 (21.83,27.2)	26.33 (23.7,29.46)	< 0.001
**hs-CRP (mg/dL)—median(IQR)**	1.42 (0.7,3.25)	0.32 (0.2,0.6)	0.29 (0.18,0.64)	1.33 (0.64,3.09)	< 0.001
**eGFR (mL/min/1.73m**^**2**^**)- median(IQR)**	88.25 (76.14,101.04)	99.18 (81.51,109.13)	106.27 (92.57,120.5)	88.98 (76.48,102.07)	< 0.001
**Poor management: Yes**	5776 (52.8)	46 (52.27)	233 (43.96)	6055 (52.39)	< 0.001
**cIMT (mm)—median(IQR)**	0.58 (0.52,0.68)	0.57 (0.49,0.7)	0.54 (0.49,0.62)	0.58 (0.52,0.68)	< 0.001

^a^ Numbers are N (%) unless noted otherwise

Abbreviations: IQR–interquartile range; AMI–acute myocardial infarction; CVD–cardiovascular disease; BMI–body mass index; DM–diabetes mellitus; HDL–high density lipoprotein; LDL–low density lipoprotein; hs-CRP high sensitivity C-reactive protein; CMV–cytomegalovirus.

**Table 2:** Specific characteristics of HIV-infected patients by cART status.

	*cART*	*Naive*	*Total*	*P value*
Total	476	59	535	
Nadir CD4 cells/mm^3^—median(IQR)	189 (84,284.5)	499.5 (427.25,617.25)	215 (97,314)	< 0.001
CD4 cells/mm^3^—median(IQR)	531 (340.5,736.5)	576 (492,680)	534 (365,735.25)	0.0485
Undetectable VL: Yes	366 (77.38)	11 (19.3)	377 (71.13)	< 0.001
Time since Diagnosis (years)–median (IQR)	9 (3.94,14.7)	4.63 (2.68,7.86)	7.89 (3.44,14.37)	< 0.001
Time of cART use (years)—median(IQR)	4.64 (1.67,10.84)	-	-	-
PI use: Yes	281 (59.03)	-	-	-
Time of PI use (years)—median(IQR)	1.02 (0,7.18)	-	-	-
NNRTI use: Yes	349 (73.32)	-	-	-
Time of NNRTI use (years)—median(IQR)	1.72 (0,4.2)	-	-	-

Abbreviations: IQR–interquartile range;PI: protease inhibitor; NNRTI–non-nucleoside reverse transcriptase inhibitor: VL: viral load

The best linear model chosen for both comparisons did not include the HIV group, but we included the variable in the final unweighted model, which confirmed that there was no significant difference when comparing HIV group to either ELSA-Brasil (Table 3) or friend controls (Table 4) after adjusting for all the variables chosen in the final model. In the weighted models (both univariate and multiple), HIV group was not significantly associated with cIMT, compared to ELSA-Brasil (Table 3) and to friend controls (Table 4). In the unweighted multiple model, several traditional CVD risk factors were also significantly associated with cIMT in both comparisons. When the ELSA-Brasil group was used as control (Table 3), protective factors were female gender, higher education and higher HDL while risk factors were older age, current and former smoker, family history of AMI or stroke, history of cardiovascular disease, hypertension, diabetes mellitus, higher BMI, and higher total cholesterol. Use of lipid-lowering drugs was kept in the model, but was not statistically significant. Hypertension was the strongest associated factor with an average increase of 0.033 mm among individuals with this diagnosis (95% CI = 0.029; 0.038, p-value<0.001). When friend controls were used as the comparison group (Table 4), older age, family history of AMI or stroke, hypertension, higher BMI and total cholesterol were significant risk factors, while female gender and use of lipid lowering drugs were protective. Point estimators are not interpretable in the multiple weighted model.

**Table 3:** Final model for cIMT compared with the ELSA-Brasil control group.

*Univariable Models*	*Unweighted*			Weighted*		
*Variable*	*Estimate*	*95%CI *	*p-value*	*Estimate*	* 95%CI*	*p-value*
Group: HIV	-0.0434	-0.0549;-0.0319	<0.01	-0.007	-0.0282;0.015	0.55
** **						
**Multivariable Models**	***Unweighted***			Weighted*		
*Variable*	*Estimate*	* 95%CI*	*p-value*	*Estimate*	* 95%CI*	*p-value*
Group: HIV	0.0038	-0.0063;0.0139	0.46	0.002	-0.0167;0.0208	0.83
Age (10 year)	0.062	0.06;0.065	<0.01	0.066	0.058;0.075	<0.01
Gender: Female	-0.0247	-0.029;-0.0203	<0.01	-0.0188	-0.0322;-0.0054	0.01
Schooling > = High School: Yes	-0.0168	-0.0227;-0.0109	<0.01	-0.0183	-0.0323;-0.0044	0.01
Smoking: former	0.0067	0.0021;0.0113	<0.01	0.0068	-0.0087;0.0223	0.39
Smoking: current	0.0257	0.0196;0.0317	<0.01	0.0087	-0.0094;0.0268	0.35
Family History of AMI or stroke: Yes	0.0109	0.0064;0.0154	<0.01	0.0254	0.0076;0.0432	0.01
History of CVD: Yes	0.0136	0.0044;0.0228	<0.01	0.02	-0.0093;0.0492	0.18
Hypertension: Yes	0.0331	0.0284;0.0378	<0.01	0.0287	0.0152;0.0422	<0.01
Diabetes Mellitus: Yes	0.0165	0.0109;0.0221	<0.01	0.0156	0;0.0312	0.05
BMI in Kg/m^2^	0.0027	0.0023;0.0032	<0.01	0.0018	0.0006;0.0031	0.01
Total Cholesterol in mg/dL	0.0003	0.0002;0.0003	<0.01	0.0003	0.0001;0.0005	<0.01
HDL in mg/dL	-0.0005	-0.0006;-0.0003	<0.01	-0.0006	-0.001;-0.0002	<0.01
Use of lipid-lowering drug: Yes	0.006	-0.0003;0.0124	0.06	-0.0024	-0.0186;0.0138	0.77

* Point estimates of weighted models are not interpretable and are shown for the sake of full data availability

Abbreviations: AMI–acute myocardial infarction; CVD–cardiovascular disease; BMI–body mass index; HDL–high density lipoprotein.

**Table 4:** Final model for cIMT compared with the Friemds control group.

*Univariable Models*	*Unweighted*			Weighted*		
*Variable*	*Estimate*	* 95%CI*	*p-value*	*Estimate*	* 95%CI*	*p-value*
Group: HIV	-0.0355	-0.0629;-0.0081	0.01	-0.016	-0.048;0.0161	0.33
** **						
**Multiple Models**	***Unweighted***			Weighted*		
*Variable*	*Estimate*	* 95%CI*	*p-value*	*Estimate*	* 95%CI*	*p-value*
Group: HIV	-0.0154	-0.0374;0.0066	0.17	-0.0113	-0.0353;0.0126	0.35
Age (10 year)	0.058	0.05;0.066	<0.01	-0.512	-0.738;-0.287	<0.01
Gender: Female	-0.0433	-0.0587;-0.0278	<0.01	0.0086	-0.0145;0.0318	0.47
Family History of AMI or stroke: Yes	0.0207	0.0043;0.0372	0.01	0.0518	0.0212;0.0824	<0.01
Hypertension: Yes	0.0465	0.0289;0.064	<0.01	0.0058	0.0045;0.0071	<0.01
BMI in Kg/m^2^	0.0026	0.001;0.0043	<0.01	0.0032	0.0012;0.0052	<0.01
Total Cholesterol in mg/dL	0.0002	0;0.0004	0.04	0.0004	0.0001;0.0007	0.01
Use of lipid-lowering drug: Yes	-0.0276	-0.0507;-0.0045	0.02	-0.0236	-0.052;0.0048	0.10

* Point estimates of weighted models are not interpretable and are shown for the sake of full data availability

Abbreviations: AMI–acute myocardial infarction; BMI–body mass index.

## Discussion

In this study, we compared cIMT values in a group of HIV-infected patients and 2 groups: a confirmed HIV-negative group of patients’ friends and the ELSA-Brasil group. To our knowledge, this is the largest study conducted in a middle-income setting describing this comparison. The three groups were very heterogeneous regarding several studied characteristics and the HIV group presented lower cIMT values, compared with the other two groups. After controlling for several CVD risk factors, cIMT was not different among all 3 groups, although it was still thicker on average for friend controls compared to HIV patients, albeit not statistically significant (Table 4). The model also confirmed several traditional risk factors associated with increased cIMT, as expected. The lack of association was confirmed by the use of a PSW technique that was able to balance data differences between groups.

Even though there is a great body of evidence in the literature pointing to a moderate association between HIV infection and increased cIMT [18], both in cross-sectional settings and in prospective studies, the results are still controversial. In a meta-analysis of observational studies, the consensus measurement revealed that HIV-infected patients had a moderate 0.04mm increase in cIMT compared with HIV-negative individuals [33]. Despite this result being confirmed in a subsequent large study [16], several of the selected studies in the meta-analysis were small and the larger studies tended to cluster around no difference between the groups [33]. In a recent large study, cIMT progression was not associated with HIV infection [34]. The baseline paper of the same study did not show association between HIV infection and cIMT either [35]. Moreover, at least two other recently published studies set in low-income countries in Africa also point to association between traditional risk factors with subclinical atherosclerosis, but not HIV-specific characteristics [36, 37]. These findings are in agreement with our recent analysis among HIV-infected patients only [21].

These apparent conflicting results may stem from several issues involving design and analysis of the data. One main problem is the fact that cIMT is not uniformly measured, despite the existence of a well described consensus, which was followed in this study [27]. As a matter of fact, the baseline analysis of cIMT of ELSA-Brasil also showed a low impact of traditional risk factors on cIMT [23], and cIMT has been recently regarded as a weak predictor of atherosclerosis at the common carotid artery and when plaque measurement is absent [38], as is the case in the current study.

Additionally, there could be peculiarities among HIV-infected patients in the sense that carotid artery segments can be affected differently in HIV-infected patients compared to non-infected individuals, e.g. internal carotid and bifurcation have more consistent association results than common carotid measurements [34]. Other issues include small studies, lack of suitable control groups, and lack of variables that could be used to control for confounding in observational [39]. All these issues should be taken into account when critically discussing cIMT-related findings.

Even though increased CVD risk among HIV-infected patients has been well established [40], one possible explanation for the lack of association between HIV status and cIMT is that specific mechanisms that lead to increased CVD risk among HIV-infected patients may not involve the traditional mechanisms described so far, including cIMT. Alternative mechanisms may include hepatitis C coinfection, low CD4 counts, high levels of viremia and other factors may lead to a chronic state of inflammation and immunological dysregulation that may explain higher CVD risk in HIV-infected individuals, acting differently from HIV-negative individuals [41].

Although our study was large and did have a multitude of variables to be used to control for possible confounding with different approaches, we did notice that HIV-infected patients seemed indeed to have a lower risk of CVD as measured by cIMT. In fact, our patients also presented a high percentage of lipid-lowering drugs usage–even higher than the ELSA-Brasil cohort–and that may be due to the timing the study was conducted, after the established awareness of physicians in respect to CVD risk among HIV-infected patients. This also denotes that in our institution high standards of care were achieved regarding HIV-infected patients’ management, which comprises a multidisciplinary approach with the participation of cardiologists and endocrinologists to closely monitor dyslipidemia and CVD. Another possibility is that HIV-infected patients would not be at risk for long enough in their natural history of disease, but 2 measurements, nadir CD4 and time since HIV diagnosis, denote that enough time has elapsed (overall nadir CD4 215 cells/mm^3^ and 7.9 years since HIV diagnosis, Table 2).

Moreover, when we tested a variable to capture clinical management of hypertension, DM and dyslipidemia, the HIV group had the lowest proportion of individuals with “poor management”: 44% vs. 52.3% and 52.8% among friend controls and ELSA, respectively (p-value <0.001, Table 1).

Even though race was not included as one of the variables to fit the best model chosen, when added to the HIV vs. ELSA-Brasil model, it was significant (beta = 0.01, p-value<0.001), but not in the model that compared HIV vs. friend controls (beta = 0.006, p-value = 0.467). The fact that race was not selected to enter the final model may be due to very little contribution to improve model fitness.

Our study has several limitations. First, this was an observational, cross-sectional study, which has all the known problems regarding indication bias in the case of treatment for CVD risk factors differentially among groups and heterogeneity among the groups. There is also the possibility of patients nominating friends that were knowingly sicker than an “average friend”, so they could have access to a full clinical evaluation they would not have in regular conditions. As any observational study, we cannot guarantee that all possible confounding was controlled for, e.g. information about HCV infection was not available for participants, and there always is the possibility of residual confounding. On the other hand, this was a very large study that had an extensive panel of variables that were used to control for those possible confounding characteristics, both with unweighted multiple linear models and also with a data balancing technique, confirming the results of no significant differences among the groups in the final model.

Another potential problem was missing values for cIMT in the study. Even though we did have about 20% of participants that did not have cIMT measured and more participants with higher risk for thicker cIMT were in this group (see Tables A-D in S1 File), this differential bias was significant in both control groups, but not in the HIV group. Thus the bias introduced was in the direction of the null hypothesis, but from the opposite direction of our hypothesis of thicker cIMT among HIV-infected patients. Since we have all other variables used to control for in the adjusted models, we don’t think that the residual bias would be significant had those participants had valid results, and thus we don’t think this would invalidate our findings.

Additionally, the HIV status for the ELSA-Brasil study participants is unknown, but given the characteristics of the sample comprised of Brazilian Federal Public workers, who tend to be healthier than the general population, we believe that HIV prevalence would be in the worst scenario the same as the overall Brazilian population, which is 0.4% [42] and would have limited impact on the comparisons presented.

In conclusion we show for the first time in a middle-income setting that cIMT, an early marker for CVD, is not different in HIV-infected patients in Rio de Janeiro compared with 2 different groups of non-HIV-infected individuals, and that traditional CVD risk factors are associated with this outcome. Our results point out that high standards of care and prevention for CVD risk factors should always be sought both in the HIV-infected population and in the general population to prevent CVD-related events.

## Supporting Information

S1 File Comparisons between participants who did and did not have valid cIMT results–overall and per group. Table A, Overall; Table B, ELSA-Brasil; Table C, Friend controls; Table D, HIV group.
(XLSX)
